# Transcriptomic Analysis Reveals Key Genes Involved in Oil and Linoleic Acid Biosynthesis during *Artemisia sphaerocephala* Seed Development

**DOI:** 10.3390/ijms22168369

**Published:** 2021-08-04

**Authors:** Shuzhen Nan, Lijing Zhang, Xiaowei Hu, Xiumei Miao, Xiaoxu Han, Hua Fu

**Affiliations:** State Key Laboratory of Grassland Agro-ecosystems, Key Laboratory of Grassland Livestock Industry Innovation, Ministry of Agriculture and Rural Affairs, Engineering Research Center of Grassland Industry, Ministry of Education, College of Pastoral Agriculture Science and Technology, Lanzhou University, Lanzhou 730020, China; nanshzh16@lzu.edu.cn (S.N.); huxw16@lzu.edu.cn (X.H.); miaoxm@lzu.edu.cn (X.M.); hanxx17@lzu.edu.cn (X.H.)

**Keywords:** *Artemisia sphaerocephala*, oil accumulation, linoleic acid, seed development, transcriptome analysis

## Abstract

*Artemisia sphaerocephala* seeds are rich in polysaccharides and linoleic acid (C18:2), which have been widely used as traditional medicine and to improve food quality. The accumulation patterns and molecular regulatory mechanisms of polysaccharides during *A. sphaerocephala* seed development have been studied. However, the related research on seed oil and C18:2 remain unclear. For this study, *A. sphaerocephala* seeds at seven different development stages at 10, 20, 30, 40, 50, 60, and 70 days after flowering (designated as S1~S7), respectively, were employed as experimental samples, the accumulation patterns of oil and fatty acids (FA) and the underlying molecular regulatory mechanisms were analyzed. The results revealed that oil content increased from 10.1% to 20.0% in the early stages of seed development (S1~S2), and up to 32.0% in mature seeds, of which C18:2 accounted for 80.6% of the total FA. FA and triacylglycerol biosynthesis-related genes jointly involved in the rapid accumulation of oil in S1~S2. Weighted gene co-expression network analysis showed that transcription factors *FUS3* and *bHLH* played a critical role in the seed oil biosynthesis. The perfect harmonization of the high expression of *FAD2* with the extremely low expression of *FAD3* regulated the accumulation of C18:2. This study uncovered the gene involved in oil biosynthesis and molecular regulatory mechanisms of high C18:2 accumulation in *A. sphaerocephala* seeds; thus, advancing research into unsaturated fatty acid metabolism in plants while generating valuable genetic resources for optimal C18:2 breeding.

## 1. Introduction

Vegetable oils are an important component of the human diet that provide energy, as well as a variety of fatty acids (FAs) required to maintain optimal health. Linoleic acid (C18:2, LA) is an essential FA that cannot be synthesized in vivo by humans and mammals; thus, it must be obtained from the diet. Simultaneously, conjugated linoleic acid (CLA) (a natural active substance with immunological functions) is generated in the rumen of ruminants using C18:2 as a substrate. C18:2 and CLA play critical roles in the prevention of cancer and various cardiovascular, inflammatory, and autoimmune diseases [1,2].

Seeds are important storage organs for vegetable oils, which are primarily stored as triacylglycerols (TAG), with variable content in different plant species. The oil content of walnut (*Juglans regia*) embryo, peanut (*Arachis hypogaea*), canola (*Brassica napus*), upland cotton (*Gossypium hirsutum*), and soybean (*Glycine max*) were found to be 69.1%, 58.2%, 42.7%, 35.2%, and 15.0~20.2%, respectively [3,4,5,6,7]. The accumulation of seed oil is a complex process, which primarily consists of two parts: FA *de novo* biosynthesis in plastids and TAG biosynthesis in endoplasmic reticulum (ER) [8,9]. 

FA *de novo* biosynthesis primarily occurs in the plastids. Acetyl-CoA carboxylase (ACCase) catalyzes acetyl-CoA to form malonyl-CoA, whereafter malonyl-CoA ACP S-malonytransferase (MAT) further transfers malonly-CoA to malonly-ACP. Subsequently, six continuous condensation reactions are catalyzed by 3-ketoacyl-ACP synthase (KAS), 3-ketoacyl-ACP reductase (KAR), 3-hydroxyacyl-ACP dehydratase (HAD) and enoyl-ACP reductase (EAR) to form palmitic acid-ACP (C16:0-ACP), which can either be converted to free C16:0 by the fatty acyl-ACP thioesterase B (FATB) or further elongated by KASII to stearic acid-ACP (18:0-ACP). The 18:0-ACP can be hydrolyzed to free C18:0 by FATB or be desaturated to oleic acid-ACP (18:1-ACP) by 18:0-ACP desaturase (SAD), whereafter 18:1-ACP is hydrolyzed to free C18:1 by the fatty acyl-ACP thioesterase A (FATA). Free fatty acid (C16:0, C18:0, C18:1) are esterified to FA-CoA by long-chain acyl-CoA synthetase (LACS) and then transported into ER [8,9]. C18:1-CoA can be incorporated into phosphatidylcholine (PC) by lysophosphatidylcholine acyltransferase (LPCAT), and in turn be dehydrogenated to C18:2-PC and linolenic acid-PC (C18:3-PC) by omega-6 desaturase (FAD2) and omega-3 desaturase (FAD3) [10,11]. 

The different characteristics of vegetable oils depend on the FA composition, 18C unsaturated fatty acid (UFAs) are important constituents of vegetable oils, and the regulatory mechanisms were also different: between species with C18:1 as the main FA component, the up-regulation of *KASII* and *SDA6* resulted in the abundant accumulation of C18:1 in Siberian apricot (*Prunus sibirica*) [12]. The perfect harmonization of high *SAD* levels with low *FAD2* levels facilitated the accumulation of C18:1 in *Camellia oleifera* and hickory (*Carya cathayensis*) [13,14]. The main UFA of Perilla (*Perilla frutescens*) and tree peony (*Paeonia* section *Moutan*) seed oil were C18:3, however, the formation of this trait was regulated by highly expressed *FAD3* and *FAD8*, respectively [15,16]. *G. hirsutum* and walnut (*Juglans regia*) with C18:2 as the main FA, the high expression of *FAD2* and the very low expression of *FAD3* were the key reasons for the formation of *G. hirsutum* C18:2 [7], while in *J. regia*, the expression level of *FAD3* was higher than that of *FAD2*, and the reason for the fact that the content of C18:3 was lower than that of C18:2 remains to be further studied [5]. The above results further indicate that the regulatory mechanism for the formation of the same FA trait varied from species to species.

TAG biosynthesis occurs in ER via two pathways: (1) Kennedy pathway (or acyl-CoA-dependent pathway), which involves three sequential acylations of acyl-CoAs to the glycerol-3-phosphate (G3P); (2) acyl-CoA-independent pathway. In the Kennedy pathway, glycerol-3-phosphate acyltransferase (GPAT) transfers a FA of acyl-CoA to the sn-1 hydroxy group of G3P to form lysophosphatidic acid (LPA). Subsequently, lysophosphatidic acid acyltransferase (LPAT) catalyzes the second acylation at the sn-2 hydroxy group of G3P to yield phosphatidic acid (PA). Next, the phosphate in the sn-3 position of the PA is removed by phosphatidic acid phosphatase (PAP), to form diacylglycerol (DAG). Finally, acyl-CoA: diacylglycerol acyltransferase (DGAT) catalyzes the acylation reaction at the sn-3 hydroxy group of DAG to produce TAG [17]. In the acyl-CoA-independent pathway, three pathways allow the flux of FA through PC for the eventual biosynthesis of TAG: (1) Exchange FA between the PC pool and acyl-CoA pool through acyl editing; (2) PDAT directly transfers a FA from the sn-2 position of PC to the sn-3 hydroxyl group of DAG to form TAG; (3) Using PC-derived DAG as substrate for TAG biosynthesis [8,17].

The preference of DGAT and PDAT for TAG biosynthesis varies with plant species. For example, DGAT is more important for the synthesis of TAG in *B. napus* [18,19,20]. Whereas in soybean, castor bean (*Ricinus communis*), sunflower (*Helianthus annuus*) and *Arabidopsis thaliana*, which primarily uses PC-mediated pathways under the action of PDAT to form TAG [21,22]. 

In addition, some transcription factors, such as *WRINKLED1* (*WRI1*), *FUSCA3* (*FUS3*), *ABSCISIC ACID INSENSITIVE* 3 (*ABI3*), and *LEAFY COTYLEDON1*, *2* (*LEC1*, *LEC2*) play key roles in seed development and oil accumulation. The mutation and overexpression of these genes have an important impact on seed development and oil accumulation [23,24].

*Artemisia sphaerocephala* is a superdry perennial semi-shrub belonging to the *Artemisia* genus of the *Compositae* family, which is a very important windproofing and sand-fixing plant on the mobile semi-stable sand dunes in the arid desert regions of Northwest China [25]. The polysaccharides of *A. sphaerocephala* seeds account for 39.8% of the dry seed weight [26], which have important physiological and ecological value [27]. It also has antioxidant, antidiabetic, anti-obesogenic, antitumor, and immunomodulatory activities, and can also be used as a food additive to improve food quality [28]. The accumulation patterns and molecular regulatory mechanisms of polysaccharides during seed development of *A. sphaerocephala* have been initially uncovered [26,29]. It is worth noting that oil content of *A. sphaerocephala* seeds account for 21.5% of the dry seed weight, of which C18:2 account for 78.6% of the total FA [30], and possess the largest *FAD2* gene family with twenty-six members in the plant kingdom [31]. However, the accumulation patterns of seed oil and C18:2 during seed development and the potential molecular regulatory mechanisms involved have not been reported to date. 

For this study, *A. sphaerocephala* seeds were employed as experimental materials, where oil and FA accumulation patterns were determined for seven different stages of seed development. Simultaneously, the molecular mechanism regulating this process were revealed via short-read next-generation RNA sequencing and third-generation single-molecule real-time sequencing. The results of this study will contribute to a deeper understanding of the molecular kinetics behind the biosynthesis of oil and the regulation of FA components in plants, while laying the foundation for the development of excellent genetic resources toward the production of high-quality oils from *A. sphaerocephala*.

## 2. Results and Discussion

### 2.1. Morphological Characteristics and Oil Accumulation during A. sphaerocephala Seed Development

**Figure 1:**
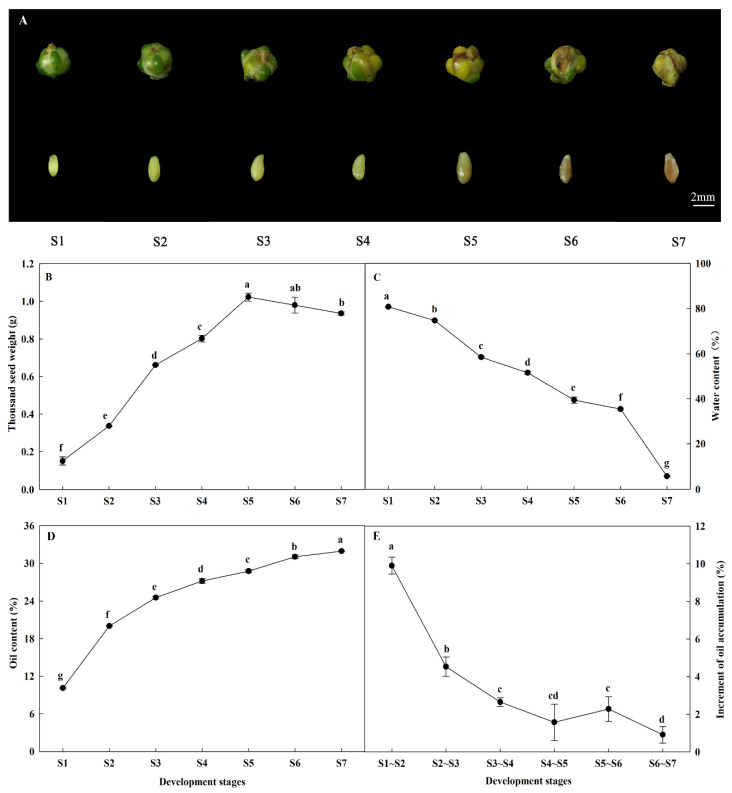
Morphological characteristics and oil content during seed development stages. (**A**) morphological characteristics; (**B**) thousand seed weight; (**C**) water content; (**D**) oil content; and (**E**) increment of oil accumulation. Note: S1~S7 represent 10, 20, 30, 40, 50, 60, and 70 days after flowering, respectively. The upper row represents the pods, and the lower row represents the seeds. Increment of oil accumulation = difference in oil content of two adjacent stages. Different lowercase letters indicate significant differences at *p* < 0.01.

The development of *A. sphaerocephala* seeds proceeded for 70 days, and seven different development stages at 10, 20, 30, 40, 50, 60, and 70 days after flowering (S1~S7) were investigated. The seed coat gradually changed in color, from light green to dark brown (Figure 1A). The thousand seed weight increased continuously, from 0.15 g at S1 to 1.02 g at S5, followed by a slight reduction to 0.94 g at maturity (Figure 1B). The water content was observed to decrease continuously, from 80.79% to 5.74% (Figure 1C). 

The content of seed oil and its accumulation patterns varied with plant species. The oil content of *A. sphaerocephala* seeds continuously increased from 10.1% to 32.0% with seed development (Figure 1D), which was higher than *G. max* (~20%) and *Zea mays* (6.7%) [3,32], but lower than *B. napus* (42.7%), *A. hypogaea* (53.8%), and sesame (*Sesamum indicum*) (41.3%~62.7%) [4,6,33]. Its relatively low oil content in *A. sphaerocephala* seeds may be related to its high polysaccharides content (39.8%) [26,27]. 

The development of seeds (from flowering to full maturity of seeds) from *P. frutescens*, *G. hirsutum*, *B. napus*, *P. sibirica*, and *Symplocos paniculata* proceeded for 35, 50, 56, 70, and 170 days, respectively. The exception was the seed oil content of *P. frutescens* and *A. sphaerocephala*, which increased continuously with seed development as the oil content of other seeds increased gradually, but then decreased slightly at maturity. The maximum increments of seed oil accumulation for the species listed above appeared mostly during the middle and late stages (14~28, 20~30, 28~42, 40~60, and 80~140 days, respectively) [4,7,12,16,34]. This was distinct from the maximum oil increment of *A. sphaerocephala* seeds during the early stage (S1~S2) (Figure 1E). That is, the highest seed oil accumulation content for *A. sphaerocephala* appeared at S1~S2 at 9.9%, whereafter the increment decreased at S2~S3, S3~S4, S4~S5, S5~S6, and S6~S7, which were 4.5%, 2.7%, 1.6%, 2.3%, and 0.9%, respectively (Figure 1E). Furthermore, S7 stage had the highest oil content and the water content of less than 10%, indicating which was optimal harvest time. It was consistent with the harvest time for in November of *A. sphaerocephala* seeds in actual production.

### 2.2. Characteristics of FA Compositions during A. sphaerocephala Seed Development

**Figure 2:**
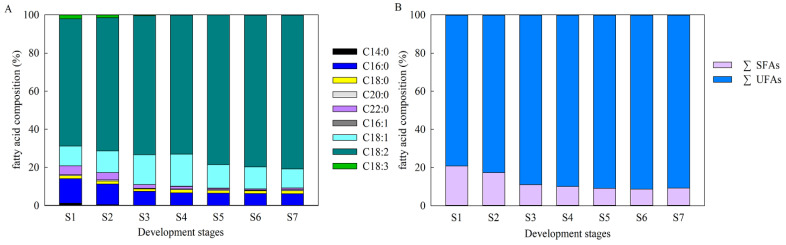
FA composition and content at different *A. sphaerocephala* seeds developmental stages (%, *w*/*w*). (**A**) Changs in the FA composition during S1~S7; and (**B**) Changs in the SFAs and UFAs during S1~S7.

As shown in Figure 2A, 18C FAs was the main component of FAs in *A. sphaerocephala* seed oil, which continuously increased from 80.8% at the early seed development stage to 92.4% in mature seeds. During S1~S7, the saturated fatty acids (SFAs) decreased from 20.8% to 9.2%, which was primarily caused by the C16:0 and behenic acid (C22:0) content decreasing from 12.9% to 6.1%, and from 4.6% to 0.9%, respectively. The UFAs increased from 79.3% to 90.8%, which was mainly due to the continuous increase of the C18:2 content, from 66.7% to 80.6%. It is worth noting that C18:2 rapidly increased from 72.9% to 78.4% from S4~S5. Changes in the SFAs and UFAs primarily occurred from S1~S3, when the SFAs content decreased from 20.8% to 11.0%, and UFAs content increased from 79.3% to 89.0% (Figure 2, Table S1).

### 2.3. Transcriptomic Analysis and Functional Annotation

Based on the RNA-Seq sequencing of 21 cDNA libraries from *A. sphaerocephala* seeds at seven different developmental stages were sequenced, a total of 166.6 Gb Clean Data was generated, with GC contents ranging from 42.52% to 45.61%, and Q30 ≥ 92.28% (Table S2). Based on the Pacific Biosciences SMRT sequencing technology, a total of 6,976,910 subreads (7.37 G base) with an average length of 1057 bp and N50 of 1149 bp were obtained. To provide more accurate sequence data, circular consensus sequences (CCS) were generated from reads that were passed at least twice through the insert, where a total of 446,687 CCS with an average length of 1042 bp were obtained. Among these, 329,538 were identified as full-length non-chimeric reads (Flnc) with an average length of 1042. Flnc were clustered using an iterative isoform-clustering algorithm, to obtain 146,134 consensus reads with an average length of 1097 bp and N50 of 1215 bp. Following error correction using the RNA-Seq data derived from the seeds of the seven different seed development stages and removal of redundant sequences via CD-Hit, a yield of 84,239 non-redundant high-quality genes was achieved (Table S3, Figure S1).

All 84,239 genes were annotated by searching NR, NT, Pfam, Swiss-Prot, TrEMBL, KOG, GO, and KEGG databases, and a total of 72,240 genes (85.76%) was annotated (Table S4). Among these, 21,525 (25.55%) genes were annotated and assigned to 118 biological pathways which involved five functional categories in KEGG database (Tables S5 and S6). In the “metabolism” category with the largest number of genes (9823, 45.64%), “energy metabolism”, “carbohydrate metabolism”, and “amino acid metabolism” were the top three enrichment pathways, with 4118 (41.92%), 3700 (37.67%), and 2046 (20.83%), respectively, followed by “lipid metabolism” with 856 (8.71%) (Table S5). In “lipid metabolism”, 168, 149, and 137 genes were enriched in “glycerophospholipid metabolism”, “glycerolipid metabolism”, and “fatty acid biosynthesis”, respectively (Table 1).

### 2.4. Identification of Candidate Genes Involved in Oil Biosynthesis

**Figure 3:**
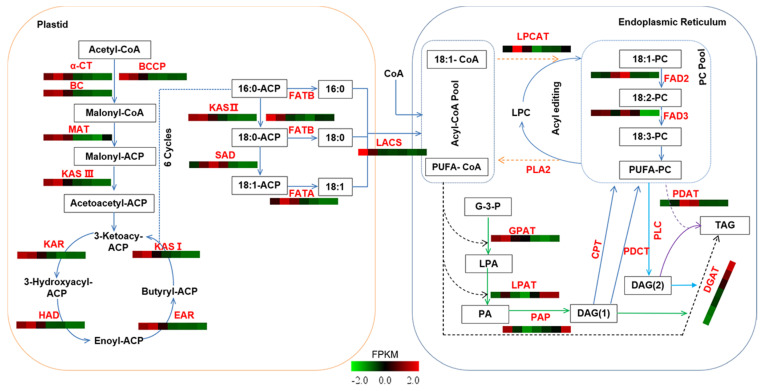
Metabolic pathways and gene expression patterns of oil accumulation during seed development of *A. sphaerocephala*. Note: The seven colored boxes in each horizontal row correspond to the seven developmental stages. The expression level of each gene in the heatmap is the sum of the FPKM of all DEGs encoding the enzyme. The color scale indicates the expression levels (represented by the log_2_FPKM) of genes. The dotted line represents acyl flux, the green line represents the Kennedy pathway (acyl-CoA dependent pathway), the purple line represents the acyl-CoA independent pathway, the orange line represents acyl editing, and the blue represents PC-derived DAG synthesis, DAG (1) represents the DAG in the *de novo* biosynthesis, and DAG (2) represents the DAG derived from the PC. Abbreviations: ACCase, acetyl-CoA carboxylase; BC, biotin carboxylase; BCCP, biotin carboxyl carrier protein; α-CT, α-carboxyltransferase; β-CT, β-carboxyltransferase; ACP, acyl carrier protein; MAT, malonyl-CoA ACP S-malonytransferase; KAS, 3-ketoacyl-ACP synthase; KAR, 3-oxoacyl-ACP reductase; HAD, 3-hydroxyacyl-ACP dehydratase; EAR, enoyl-ACP reductase; SAD, stearoyl-ACP desaturase; FAT, fatty acyl-ACP thioesterase; LACS, long chain acyl-CoA; GPAT, glycerol-3-phosphate acyltransferase; PDAT, phospholipid: diacylglycerol acyltransferase; LPAT, 1ysophosphatidic acid acyltransferase; DGAT, acyl-CoA: diacylglycerol acyltransferase; PDCT, phosphatidylcholine:diacylglycerol cholinephosphotransferase; CPT, diacylglycerol cholinephosphotransferas; LPCAT, lysophosphatidylcholine acyltransferase; PLA2, phospholipase A2; PAP, phosphate phosphatase; FAD2, omega-6 desaturase; FAD3, omega-3 desaturase; G-3-P, Glycerol-3-phosphate; LPA, Lyso-phosphatidic acid; PA, Phosphatidic acid; PC, phosphatidylcholine; LPC, lysophosphatidylcholine; DAG, 1,2-Diacylglycerol; TAG, Triacylglycerol.

Based on the KEGG pathway enrichment analysis in PacBio full-length transcriptome sequencing of *A. sphaerocephala*, a biosynthesis pathway of seed oil was constructed (Figure 3). Detailed information on each gene sequence, annotation, and FPKM value are listed in Table S7. and the heat map of a single gene during seed development is shown in Figure S2.

### 2.5. Regulatory Mechanisms of High Oil Accumulation during Early Seed Development

**Figure 4:**
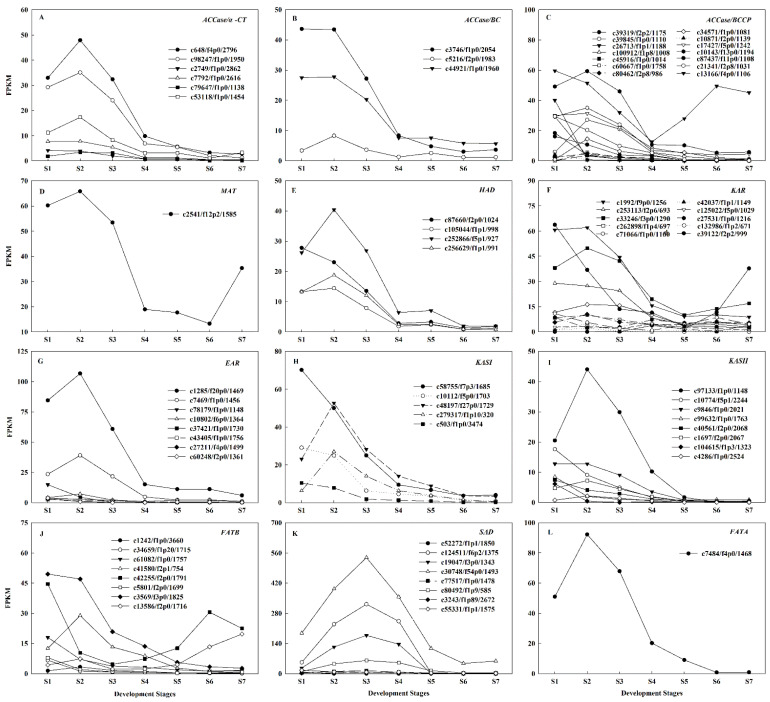
Gene expression analysis of fatty acid (FA) *de novo* biosynthesis pathway. Gene expression patterns of *ACCases*/*α-CT* (**A**), *ACCases*/*BC* (**B**), *ACCases*/*BCCP* (**C**), *MAT* (**D**), *HAD* (**E**), *KAR* (**F**), *EAR* (**G**), *KASI* (**H**)*, KASII* (**I**), *FATB* (**J**), *SAD* (**K**), and *FATA* (**L**) in developing *A. sphaerocephala* seeds.

FA *de novo* biosynthesis begins with the conversion of acetyl-CoA to malonyl-CoA, which is catalyzed by acetyl-CoA carboxylase (ACCase), comprised of four subunits, including α-carboxytransferase (α-CT), β-carboxytransferase (β-CT), biotin carboxylase (BC), and biotin carboxyl carrier protein (BCCP). Subsequently, malonyl-CoA is transferred to the malonyl group by malonyl-CoA ACP S-malonyltransferase (MAT), which is the primary substrate for the subsequent elongation. Next, 3-ketoacyl-ACP reductase (KAR), 3-hydroxyacyl-ACP dehydratase (HAD), enoyl-ACP reductase (EAR), and 3-ketoacyl-ACP synthase I (KASI) are responsible for carbon chain extension. Following six condensation cycles, the 16:0-ACP is produced. In this study, the key genes involved in fatty acid *do novo* biosynthesis demonstrated similar expression patterns. There were twenty-three *ACCases* (including six *α-CTs*, three *BCs*, and fourteen *BCCPs*), one *MAT*, ten *KARs*, four *HADs*, eight *EARs*, and five *KASIs*, all of which had the high expression levels from S1~S2, total FPKM were 87~115, 74~79, 285~237, 60~65, 224~204, 80~96, 139~164, and 138~162, respectively. This was followed by a dramatic decrease and tended to be stably expressed (Figure 4A–H). The high expression of these genes during S1~S2 were synchronized with the rapid accumulation of oil (Figure 1D), and, in turn, resulted in a significant increase of UFAs (Figure 2B). This may partially explain why oil content rapid accumulation in these early stages, which was similar to the studies of *C. cathayensis* and *C. oleifera* [13,14]. KASII is a key enzyme that controls the 16C:18C FAs ratio, whose expression is directly proportional to the content of 18C FAs [7]. The eight *KASIIs* showed relatively high total expression levels for S1~S2, FPKM ranging from 78.49 to 81.88 (Figure 4I), which formed up to 92.4% of the 18C FAs (Table S1), which was consistent with the results for *P. ostii* seeds [15].

FATB catalyzes C16:0-ACP and C18:0-ACP to form SFAs. SAD desaturates C18:0-ACP to form C18:1-ACP, which is a substrate that facilitates the formation of UFAs. The total FPKM of eight *FATBs* decreased rapidly from 145 at S1 to 48 at S3 (Figure 4J). The total FPKM of eight *SADs* increased rapidly from 307 at S1 to 1128 at S3, of which the FPKM of four *SADs* (c30748/f54p0/1493, c124511/f6p2/1375, c19047/f3p0/1343 and c80492/f1p9/585) accounted for 90.18%~97.47% of the total expression (Figure 4K). This was consistent with the significantly reduced of C16:0 and C18:0 content, from 12.9% and 1.5% to 7.3% and 1.3%, respectively, and 18C UFAs increased from 79.3% to 88.9% (Table S1). The above results implied that the rapid decline of *FATB* and the high expression of *KASII* and *SAD* generated sufficient C18:1-ACP, which was further used for the biosynthesis of 18C UFAs. Then, FATA hydrolyzes C18:1-ACP to free C18:1. The expression level of only one *FATA* gradually decreased following the attainment of a peak of 92.21 at S2 (Figure 4L), which was mostly consistent with the increase of C18:1 content, from 10.52% at S1 to 15.59% at S3, and then decreased from S4~S7 (Figure 2A, Table S1).

Seed oils are primarily stored in the form of TAG, and synthesized by acyl CoA dependent (Kennedy pathway) and independent pathways. (1) In the acyl-CoA-dependent pathway, GPAT, LPAT, PAP, and DGAT participate in the biosynthesis of TAG with glycerol-3-phosphate and acyl-CoA as substrates; (2) In the acyl-CoA-independent pathway, some acyl-CoAs will enter the PC pool and be esterified to form acyl-PCs by LPCAT, and then separate into three pathways: (1) released into the acyl-CoA pool to participate in the acyl-CoA dependent pathway; (2) synthesized TAG through PDAT; (3) converted to TAG through DAG by PDCT and CPT [8,17]. 

**Figure 5:**
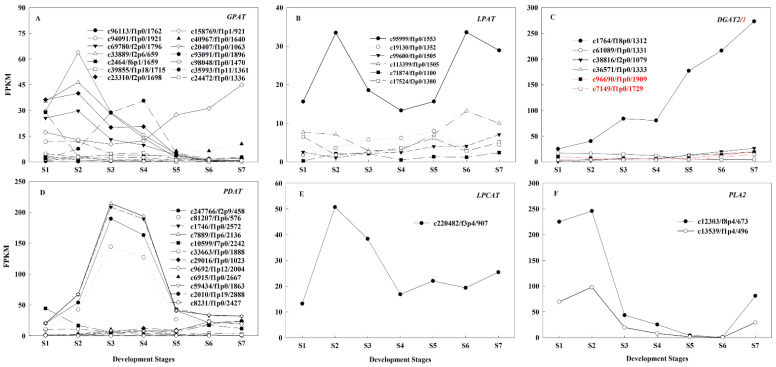
Analysis of gene expression related to triacylglycerols (TAG) biosynthesis pathway. Gene expression patterns of *GPAT* (**A**), *LPAT* (**B**), *DGAT* (**C**), *PDAT* (**D**), *LPCAT* (**E**), and *PLA2* (**F**) during *A. sphaerocephala* seed development. Note: The red in (**C**) indicates *DGAT1*.

In the acyl-CoA dependent pathway, the expression patterns of fourteen *GPATs* were diverse. Five *GPATs* (c94091/f1p0/1921, c33889/f2p6/659, c23310/f2p0/1698, c69780/f2p0/1796, and c39855/f1p18/1715) exhibited higher expression levels during S1~S2, FPKM ranging from 12 to 64, and then significantly decreased. The expression level of *GPAT* (c35993/f1p11/1361) decreased significantly after attaining a peak of 36 at S4. The *GPAT* (c158769/f1p1/921) was stably expressed from S1~S4, which FPKM continuously increased from 12 at S4 to 45 at S7 (Figure 5A). The total FPKM of the six *LPATs* ranged from 34 to 58, of which FPKM of *LPAT* (c95999/f1p0/1553) was higher (13 to 33), while the remaining DEGs expression levels were relatively low (Figure 5B). In the acyl-CoA independent pathway, one *LPCAT* and two *PLA2s* were highly expressed in S2, with total FPKM were 51 and 344, respectively (Figure 5E,F). In summary, the high expression of five *GPATs*, one *LPAT*, one *LPCAT* and two *PLA2s* from S1~S2 were synchronized with the rapid accumulation of oil (Figure 1D), speculating that they were jointly involved in the rapid accumulation of oil at these early stages. Simultaneously, the high expression of *PLA2* caused more PUFA-PC to be transferred to the acyl-coA pool, which, in turn, increased the flux of acyl-CoA-dependent pathways.

DGAT and PDAT are the key enzymes responsible for TAG biosynthesis in the acyl-CoA-dependent and acyl-CoA-independent pathways, respectively, and the relative contribution to TAG biosynthesis varied by species: *PDAT* had higher expression or higher correlation with seed oil content than *DGAT* in safflower (*Carthamus tinctorius*), *J. regia*, *G. hirsutum*, *Torreya grandis* and *P. sibirica*, indicating it may play a more important role in TAG biosynthesis [5,7,12,35,36]. Whereas in *B. napus,* DGAT was more important for the biosynthesis of TAG [18,19,20]. In this study, there was six *DGATs*, the expression level of *DGAT* (c1764/f18p0/1312) was significantly higher than that of other members, accounting for 41.93~80.50% of the total *DGATs* expression, and highly expressed from S5~S7 (Figure 5C), where phylogenetic analysis revealed that it was *DGAT2* (Figure S3). Twelve *PDATs* were found, and the expression levels of four *PDATs* (c247766/f2p9/458, c81207/f1p6/576, c1746/f1p0/2572 and c7889/f1p6/2136) were significantly higher than the others, accounting for 53.54~94.30% of the total expression, and highly expressed from S1~S3 (Figure 5D). Four main *PDATs* exhibited higher expression levels during S3 and S4, while one main *DGAT2* was highly expressed at S5 to S7 (Figure 5C,D), implying that TAG assembly mainly occurred in the middle and late stages, and these two pathways synergistically regulated TAG biosynthesis in *A. sphaerocephala* seeds. Our results agreed with those of previous studies in *C. cathayensis*, *perilla*, and *Styrax tonkinensis* seeds [13,37,38]. Meanwhile, this was also consistent with the result that the high expression of *PLA2* in this study initiated an increase in the flux of the acyl-CoA-dependent pathway. Furthermore, this study did not find the DEG encoding PDCT and CPT, which signified that this pathway did not influence the biosynthesis of TAG during *A. sphaerocephala* seed development, which was different from the results for *perilla* [37].

### 2.6. Regulation Mechanism of High C18:2 Content in Seed Oil

The C18:1, C18:2, and C18:3 content of *A. sphaerocephala* seeds accounted for 10.0%, 80.6%, and 0.1% of the total FAs, respectively (Table S1). FAD2 and FAD3 catalyze the conversion of C18:1 to C18:2 and C18:2 to C18:3, respectively [10,11]. 

**Figure 6:**
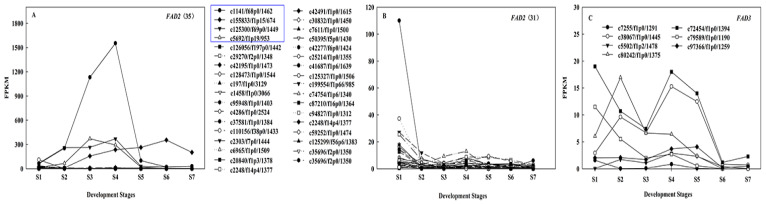
Analysis of gene expression related to FA desaturation pathway in ER. (**A**) Gene expression patterns of thirty-five DEGs encoding FAD2; (**B**) Gene expression patterns of the other thirty-one *FAD2s* genes, except for four *FAD2s* with the highest expression levels (blue boxes); (**C**) Gene expression patterns of seven *FAD3*.

During *A. sphaerocephala* seed development, there were thirty-five *FAD2s,* in which the homology of the pairwise sequence alignment was < 99% (Table S8) and seven *FAD3s*, respectively, where the total expression level of *FAD2* was 11.93~218.01 times that of *FAD3* (Figure 6). This indicated that the high expression of *FAD2* in conjunction with the low expression of *FAD3* resulted in the accumulation of C18:2 in *A. sphaerocephala* seeds. This was basically consistent with the mechanism of C18:2 accumulation in *G. hirsutum* seeds [7]. The main FA of *J. regia* was also C18:2, the high expression of *FAD3* and *FAD2* was the main reason for the enrichment of polyunsaturated fatty acids, but the expression of *FAD3* was higher than that of *FAD2*, and the mechanism of high C18:2 content remains to be further studied [5]. 

Among thirty-five *FAD2s*, four *FAD2s* (c1141/f68p0/1462, c125300/f69p0/1449, c5692/f1p19/953, and c155833/f1p15/674) were highly expressed, where the total FPKM of the four *FAD2s* increased from 145 at S1 to 2451 at S4, and then decreased to 265 at S7 (Figure 6A), which likely played a critical role in the increase of the C18:2 content, from 66.7% at S1 to 72.8% at S4, and then increased to 80.6% at S7 in *A. sphaerocephala* seeds (Figure 6A, Table S1). The other thirty-one *FAD2s* were expressed primarily at S1 (0.41~37.21), after which their expression level declined to a stable low level (FPKM < 6), presumably having a certain effect on the high content of C18:2 at S1 (Figure 6B). It is worth noting that the expression levels of other *FAD2s* were lower at S6, while the expression level of one *FAD2* (c155833/f1p15/674) reached a peak at this stage (Figure 6B), which may play a major role in the continuous conversion of C18:1 to C18:2 during S4~S7.

### 2.7. Regulation of Transcription Factors on Oil Accumulation

**Figure 7:**
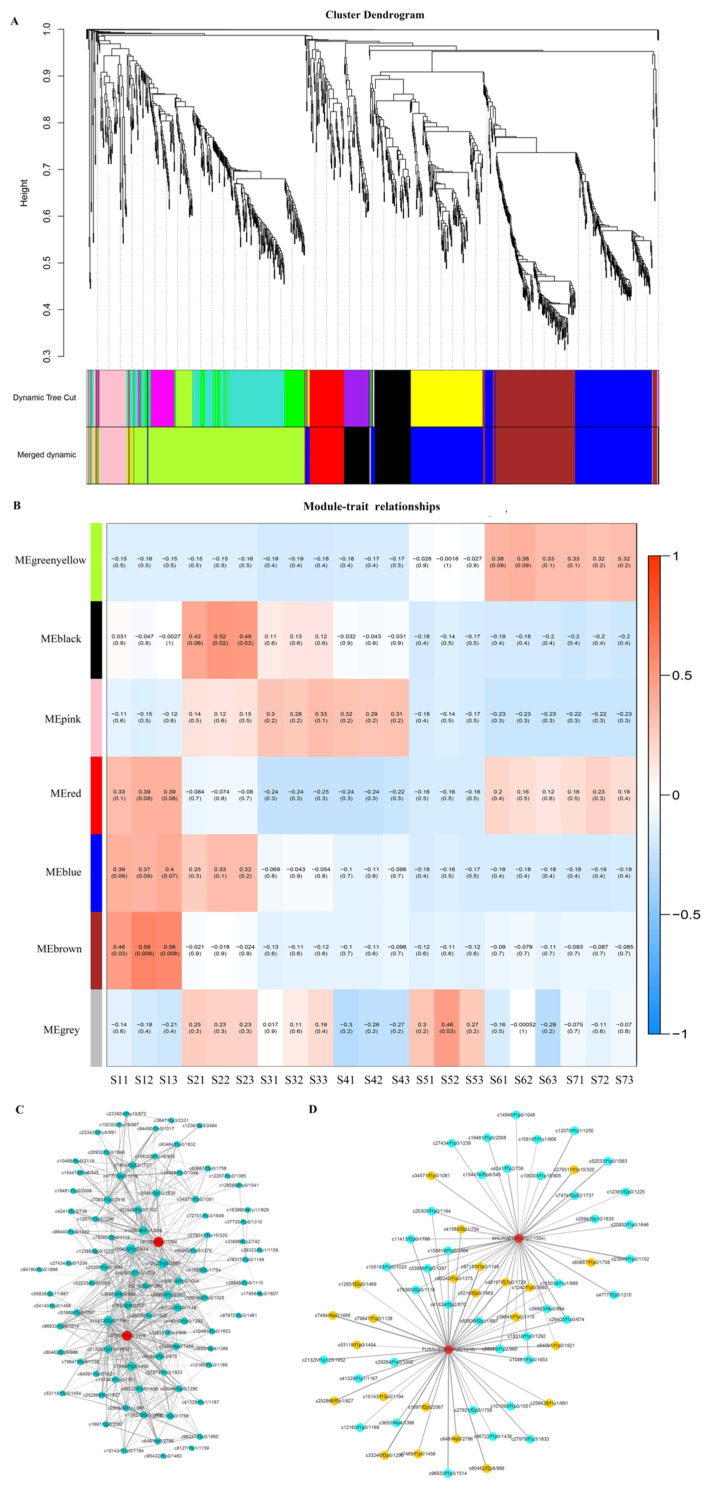
Weighted gene co-expression network analysis (WGCNA) of genes during the seed development process. (**A**) Gene co-expression modules detected by WGCNA. The clustering dendrogram of the genes across all the samples exhibits dissimilarity based on topological overlap, together with the original module colors (dynamic tree cut) and assigned merged module colors (merged dynamic). (**B**) Module-trait relationships using WGCNA. Each column corresponds a specific stage and each row corresponds to a module eigengene. Each cell contains the corresponding correlation (top number) and *p*-value (bottom number). (**C**) Co-expression network between TFs and oil biosynthesis-related genes in black module. Red points represented hub genes. (**D**) Primary co-expression network for hub genes for *FUS3* (c97806/f1p0/1418) and *bHLH* (c61080/f1p1/1304). Blue points represent gene, yellow points represent TF. The edge width represents the weight value between the two nodes: the higher the value of the weight between the nodes, the wider the edge.

Transcription factors (TFs) played a crucial role in the regulation of FA biosynthesis and the accumulation of oil in plant seeds. The key TFs that regulated the biosynthesis of oil varied by species and different organizations: *GRF5*, *WRI1*, *FUS3* were hub TFs in the oil biosynthesis regulatory network in *B. rape* seed [39]. *WRI1*, *MYB* and *ZIP* played key roles in the biosynthesis of oil in *C. oleifera* [40]; *PBS* and *RAP* played a critical role in the oil biosynthesis regulatory network in avocado (*Persea americana*) mesocarp and seed, respectively [41]. For this study, a total of 1440 differentially expressed TFs belonging to 83 different gene families were identified from the seed transcriptome of *A. sphaerocephala* at different developmental stages. Among them, the most abundant TF families were *AP2-ERF-ERF* (115), *NAC* (109), *bZIP* (73), *C3H* (66), and *bHLH* (63) (Table S9). The genes involved in the biosynthesis of FA and TAG, and TFs were subjected to weighted gene co-expression network analysis (WGCNA), and seven modules were identified and labeled by different colors (Figure 7A and Figure S4A). The seed oil content of *A. sphaerocephala* accumulated rapidly in S1~S2. The analysis of module-trait correlation relationships showed that the black module had the highest correlation with S2 (r > 0.4, *p* < 0.05), and the genes in this module were highly expressed in S2 (Figure 7B and Figure S4B), which indicated that this module related to the oil content of *A. sphaerocephala*. The black module contained 88 TFs, where the most abundant TF families were *bHLH* (10), *AP2/ERF-ERF* (8), *bZIP* (6), *AUX/IAA* (5), *C2H2* (4), and *B3* (4) (Figure S4C). Gene co-expression analysis of this module showed that the TFs *FUS3* (c97806/f1p0/1418) and *bHLH* (c61080/f1p1/1304) were the hub genes of this module (Figure 7C). Gene expression analysis revealed which genes had similar expression trends with the genes involved in oil biosynthesis, that is, had the highest expression level at S2 (Figure S4D). Moreover, *FUS3* (c97806/f1p0/1418) and *bHLH* (c61080/f1p1/1304) co-expressed with genes involved in FA biosynthesis, such as *α-CT*, *BC*, *BCCP*, *HAD*, *EAR*, *KAR*, *KASII*, *FATA*, and *FATB* (Figure 7D). The above results indicated that *FUS3* and *bHLH* were the key TFs for the accumulation of oil in *A. sphaerocephala* seeds.

### 2.8. Validation of RNA Sequencing Results by RT-qPCR

**Figure 8:**
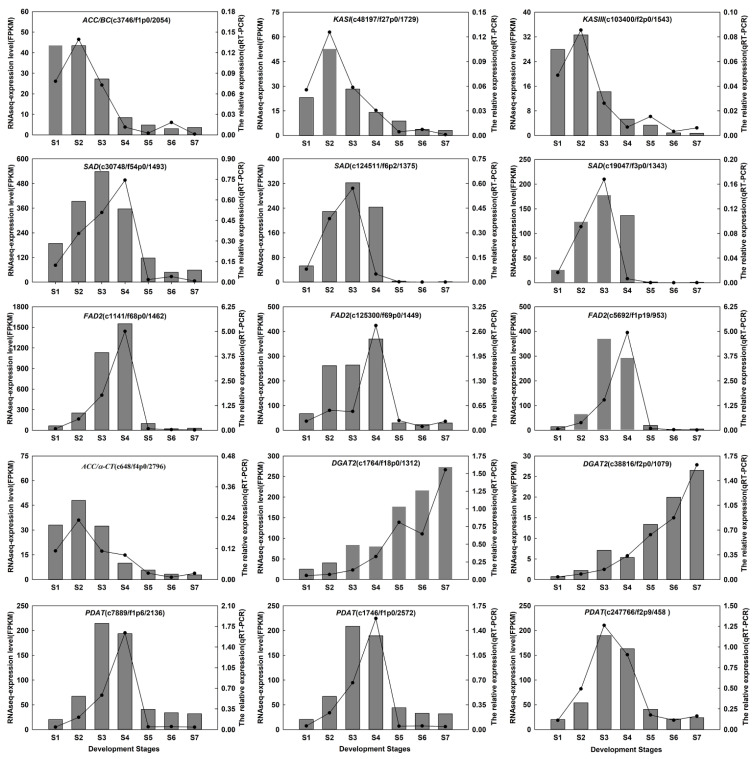
qPCR validation of fifteen candidate genes associated with oil biosynthesis during *A. sphaerocephala* seed development.

Fifteen genes associated with oil biosynthesis, including *ACCase*, *KAS*, *SAD*, *FAD2*, *FAD7*, *DGAT2*, and *PDAT* were selected for RT-qPCR validation (Table S10). The results were basically consistent with RNA sequencing (Figure 8), which indicated that the sequencing results were accurate, and the analysis of DEGs in this study was reliable.

## 3. Materials and Methods

### 3.1. Plant Materials

*Artemisia sphaerocephala* seeds were collected in their natural habitat during the seed development period (late August to mid-November 2016) in the Alxa Desert of Inner Mongolia, Northwest China (N: 38°68′, E: 105°61′). Seed pods were harvested in all plant sides, beginning on the 10th day after flowering (DAF) until full maturity, with a ten days interval between each harvest, where finally, seeds from seven different development stages were collected and designated S1 to S7. Seeds from every four plants were uniformly combined to form a single sample, with a total of three repeats. Following the removal of the bran, the seeds (about 40% of the total pods weigh at maturity) were immediately frozen in liquid nitrogen and stored at −80 °C for further use.

### 3.2. Oil Content Determination and Fatty Acid Analysis

The oil content of the seeds was determined according to the Chinese national standard method (GB 5009.6-2016). Briefly, seeds were oven-dried at 65 °C to constant weight, and then ground into powder. The total oil was extracted from 0.2 g dried powder (W_0_) at 62.5 °C for 6 h with petroleum ether as a solvent using SZC-C Fat Analyzer (Shanghai Fiber Inspection Instruments Co. Ltd., Shanghai, China). The residue was then dried at 105 °C under vacuum for 2 h and weighed (W_1_). The oil content was expressed as a percentage of seed oil to dry seed weight, and the calculation formula was as follows: % = (W_0_ − W_1_)/W_0_ × 100%.

Fatty acid methyl esters (FAMEs) were processed, after which its fatty acid composition in oil was analyzed using a GC-MS (6890N-5975C, Agilent Technologies, Santa Clara, CA, USA) equipped with a DB-FFAP chromatography column (30 m × 0.25 mm × 0.5 μm) [42]. Gas chromatography conditions: the carrier gas was high purity helium, purity ≥ 99.999%, the flow rate of the column was 1.00 mL/min. The injector temperature was 200 °C, the shunt ratio was 100:1, and the injection volume was 0.2 μL. The initial temperature of the column temperature was 70 °C, which was heated to 190 °C at 15 °C per minute, maintained for 2 min, and then heated to 230 °C at 5 °C/min and maintained for 12 min. Mass spectrometry conditions: EI ion source, the ion source temperature was 230 °C, GC-MS interface temperature was set at 250 °C, electron energy was 70 eV, and the solvent delay time was 1.5 min. The FAMEs were identified by comparing their peak retention times with those of known standards (Sigma-Aldrich, Shanghai, China), and the FA content was calculated using an area normalization technique.

### 3.3. RNA Extraction and Assessment

RNA was extracted using the Plant RNA Kit (OMEGA Bio-Tek, Norcross, GA, USA) according to the manufacturer’s instructions. Subsequently, the RNA degradation and contamination were assessed via 1% agarose gel electrophoresis, whereas the RNA purity was quantified using a Nanodrop ND1000 (Thermo Scientific, Waltham, MA, USA) (OD 260/280). The RNA concentration was measured using the Qubit RNA Assay Kit and a Qubit 2.0 Fluorometer (Life Technologies, Carlsbad, CA, USA). Further, the RNA integrity (RIN ≥ 9) was assessed using an Agilent Bioanalyzer 2100 system (Agilent Technologies, Santa Clara, CA, USA). 

### 3.4. Illumina Transcriptome Library Preparation, Sequencing, and Data Analysis

For Illumina sequencing, twenty-one RNAs were enriched with oligo (dT) magnetic beads, and randomly fragmented through the addition of a fragmentation buffer. First-strand cDNA was then synthesized with random hexamers, with fragmented mRNA being employed as a template. Second-strand cDNA was synthesized following the addition of the buffer, dNTPs, RNase H, and DNA polymerase I. The cDNA was subsequently purified with AMPure XP beads. The purified double-stranded cDNA was subjected to end repair, the addition of a poly-A tail, and ligation with sequencing linkers, and the fragment size was selected via AMPure XP beads. Finally, the cDNA library was prepared by PCR-based enrichment. After passing library quality tests, sequencing was performed using an Illumina Hiseq 2500 platform (Illumina, San Diego, CA, USA) with a 150 bp paired-end sequencing length.

Raw data in the fastq format were initially processed using internal Perl scripts. For this step, high-quality clean data were obtained through the removal of adaptor sequences and low-quality reads. Meanwhile, the quality of these clean data was estimated using the content parameters of Q30 and the GC. 

### 3.5. PacBio Iso-Seq Library Construction, Sequencing, and Data Analysis

Equal amounts of total RNA from all 21 samples were equally pooled together to prepare the Iso-Seq library. RNA was reverse-transcribed into cDNA using the SMARTer™ PCR cDNA Synthesis Kit (Clontech Laboratories Inc., Palo Alto, CA, USA). Following the PCR amplification of the enriched cDNA, we performed cDNA fragment screening using the BluePippin Size Selection System protocol and PCR amplification to enrich the full-length cDNA. Subsequently, the synthesized cDNA was subjected to end-repair, ligation with the SMRT dumbbell-shaped linker, and exonuclease digestion to obtain the library. Further, a Qubit 2.0 fluorometer and Agilent 2100 bioanalyzer were employed to confirm accurate quantification and the library size, respectively. Finally, the library was sequenced using the Pacific Biosciences RSII (Pacific Bioscience, Menlo Park, CA, USA) platform to obtain the full-length transcriptome.

Once the sequencing was completed, the raw reads were processed using SMRT Analysis software (version 2.3.0, http://www.pacb.com/products-andservices/analytical-software/smrt-analysis/, accessed on 12 December 2017). The raw polymerase reads were initially partitioned into sub-reads. A circular consensus sequence (CCS) was generated from subread BAM files, parameters: Min passes = 1, Min predicted accuracy = 0.8. Subsequently, CCS reads were classified into full-length non-chimeric (FLNC) reads, full-length chimeric reads, non-full-length reads, and short reads according to the 5′ primer-, 3′ primer-adapters and polyA tail signals; only the CCS reads with all three elements were classified as FLNC. The Iterative Clustering for Error Correction (ICE) method [43] was used to obtain consensus isoforms, and high-quality polished consensus reads were acquired from the original consensus reads corrected with the nFLNC reads.

### 3.6. Transcriptome Sequence Correction and De-Redundancy

All the isoforms were corrected using Illumina short reads with the Long-Read de Bruijn Graph Error Correction (LoRDEC) tool (http://atgc.lirmm.fr/lordec, accessed on 16 December 2017) [44]. Finally, the redundancies were removed using CD-HIT (version 4.8.1, http://weizhongli-lab.org/cd-hit/, accessed on 16 December 2017) [45] with a sequence similarity of more than 99% to obtain non-redundant high-quality transcripts. 

### 3.7. Functional Annotation and Enrichment Analysis

The DEGs sequences were aligned with the NR, Swiss-Prot, GO, COG, KOG, and KEGG databases, using BLASTX program (http://www.ncbi.nlm.nih.gov/BLAST/, accessed on 20 December 2017) [46] with cutoff E-value ≤ 10^−5^ to obtain the amino acid sequences of the DEGs. The HMMER software (E-value ≤ 10^−10^) was compared with the Pfam database to obtain the DEGs annotation information. The GO functional enrichment and KEGG pathway enrichment analyses were implemented via the GOseq R package (version 1.20.0, The Walter and Eliza Hall Institute of Medical Research, Parkville, Australia) on the basis of Wallenius noncentral hypergeometric distribution and KOBAS software (version 2.0, http://kobas.cbi.pku.edu.cn/home.do, accessed on 26 December 2017), respectively [47,48]. GO and KEGG pathways were determined to be over-represented using the Fisher exact test with an adjusted false discovery rate (FDR) correction (FDR ≤ 0.01) [49]. 

### 3.8. Quantification of Gene Expression Levels and Identification of DEGs

The expression levels were calculated by RESM (version 1.3.0, http://deweylab.biostat.wisc.edu/rsem/, accessed on 28 December 2017) and normalized by the fragments per kilobase of transcript per million mapped reads (FPKM) values [50]. FDR were used as key indicators for screening DEGs and obtained by correcting the *p*-value using the Benjamini-Hochberg correction method of hypothesis testing. Differential expression analysis was performed using the DESeq2 R package (version 1.10.1, http://bioconductor.org/packages/stats/bioc/DESeq2/, accessed on 28 December 2017) with a model based on negative binomial distribution [51], with a false discovery rate of ≤ 0.01 and a fold-change ≥ 2 as screening criteria. 

### 3.9. Quantitative Real Time PCR (RT-qPCR) Analysis

The total RNA extraction and assessment were conducted as described in Section 3.3. First-strand cDNA was synthesized using 1 μg total RNA using Prime-Script^TM^ RT reagent Kit with gDNA Eraser (Perfect Real Time) (Takara Biotechnology Inc., Dalian, China) following the manufacturer’s protocol. Gene expression levels were determined by RT-qPCR using a SYBR *Premix Ex Taq* Kit (Takara Biotechnology Inc., Dalian, China) on a QuantStudio 5 Real-Time PCR System (Applied Biosystems, Foster City, CA, USA). The 10 μL RT-qPCR reaction mixtures comprised of 5 μL of SYBR Premix Ex Taq II, 1 μL of cDNA, 0.4 μL of each specific primer, 0.2 μL of ROX Reference Dye II, and 3 μL of DEPC-treated water. The reaction conditions were as follows: 95 °C for 30 s, followed by 40 cycles of 95 °C for 5 s, and then 60 °C for 34 s. The reactions were carried out in triplicate, where *UBC9* was employed as the reference gene [52]. The relative expression levels of the genes were calculated using the 2^−ΔΔCt^ method, with the gene primers listed in Table S10.

### 3.10. Data Analysis

Heatmaps were performed using MEV 4.9 (https://sourceforge.net/projects/mev-tm4/files/mev-tm4/, accessed on 12 July 2020). For phylogenetic analysis, the unrooted phylogenetic tree was constructed using MEGA 7.0 (Arizona State University, Tempe, AZ, USA) with the neighbor joining method, and bootstrap values from 1000 replicates were indicated at each branch. The comparison of amino acid sequences was analyzed using DNAMAN 8.0, and TFs were predicted and classified into different families using the PlantTFDB 5.0 (http://planttfdb.gao-lab.org/, accessed on 1 April 2021). The gene co-expression network was constructed using WGCNA of MBKCloud platform (http://www.biomarker.com.cn/biocloud, accessed on 14 April 2021), the network diagram was drawn by Cytoscape 3.8.0 (https://cytoscape.org/, accessed on 18 April 2021), and the hub genes were identified according to the Degree Centrality algorithm of the plug-in CentiScaPe (version 2.2, Cytoscape App Store-CentiScaPe) in Cytoscape.

Data were subjected to one-way analysis of variance (ANOVA) using SPSS 17.0 (SPSS Inc, Chicago, IL, USA). Duncan’s multiple range tests were employed to determine differences at a significance level of *p* < 0.01.

## 4. Conclusions

In summary, the content of seed oil in *A. sphaerocephala* continuously increased from 10.1% to 32.0% with seed development and showed the highest accumulation from 10 days to 20 days after flowering. The high expression of FA de novo biosynthesis-related genes, encompassed *ACCase*, *MAT*, *KAS*, *KAR*, *EAR*, *HAD*, and TAG biosynthesis-related genes, including *GPAT*, *LPAT*, *LPCAT*, *PLA2* during S1~S2, which was crucial for the rapid accumulation of oil in the early seed development stages. The relatively high expression of *KASII* in S1~S2 and the rapid decrease of *FATB* resulted in the 18C FA content as high as 92.4% at this stage. The high expression of *FAD2* and low expressions of *FAD3* resulted in C18:2 accounting for 80.6% of the total FAs. Furthermore, *PDAT* and *DGAT* concurrently regulated the biosynthesis of TAG in *A. sphaerocephala* seeds. WGCNA showed that transcription factors *FUS3* and *bHLH* played a crucial role in the oil biosynthesis of *A. sphaerocephala* seeds. This study revealed the molecular regulatory mechanisms of the oil accumulation process in *A. sphaerocephala* seeds, while providing abundant genetic resources for the molecular breeding of oil crops.

## Figures and Tables

**Table 1:** Pathways and numbers of genes associated with classification of lipid metabolism.

Detabase	Pathway Level			Number (Percentage (%))
KEGG				21,525 (25.55%)
	metabolism			9823 (45.64%)
		Lipid metabolism		856 (8.71%)
			Glycerolipid metabolism	149
			Glycerophospholipid metabolism	168
			Sphingolipid metabolism	48
			Steroid biosynthesis	78
			Ether lipid metabolism	39
			Synthesis and degradation of ketone bodies	25
			Fatty acid biosynthesis	137
			Biosynthesis of unsaturated fatty acids	293
			Arachidonic acid metabolism	52
			alpha-Linolenic acid metabolism	46
			Linoleic acid metabolism	1

Note: Number represents the number of genes annotated to the database or metabolic pathway, and the percentage in parentheses represents that the percentage of this genes number in metabolic pathway level to the genes number annotated to the upper-level pathway.

## Data Availability

Raw sequence data from this study have been deposited in the NCBI Short Read Archive database (SRA) (https://www.ncbi.nlm.nih.gov/sra/, accessed on 19 June 2020) under accession number PRJNA638527.

## Supplementary Materials

The following are available online at https://www.mdpi.com/article/10.3390/ijms22168369/s1.

## Abbreviations

ACCase	acetyl-CoA carboxylase
BC	biotin carboxylase
BCCP	biotin carboxyl carrier protein
α/β-CT	α/β-carboxyltransferase
ACP	acyl carrier protein
MAT	malonyl-CoA ACP S-malonytransferase
KAS	3-ketoacyl-ACP synthase
KAR	3-ketoacyl-ACP reductase
HAD	3-hydroxyacyl-ACP dehydratase
EAR	enoyl-ACP reductase
SAD	stearoyl-ACP desaturase
FAT	fatty acyl-ACP thioesterase
LACS	long chain acyl-CoA
GPAT	glycerol-3-phosphate acyltransferase
PDAT	phospholipid: diacylglycerol acyltransferase
LPAT	1ysophosphatidic acid acyltransferase
DGAT	acyl-CoA: diacylglycerol acyltransferase
PDCT	phosphatidylcholine:diacylglycerol cholinephosphotransferase
CPT	diacylglycerol cholinephosphotransferas
LPCAT	lysophosphatidylcholine acyltransferase
PLA2	phospholipase A2
PAP	phosphate phosphatase
FAD2	omega-6 desaturase
FAD3	omega-3 desaturase
G-3-P	Glycerol-3-phosphate
LPA	Lyso-phosphatidic acid
PA	Phosphatidic acid
PC	phosphatidylcholine
LPC	lysophosphatidylcholine
DAG	1,2-Diacylglycerol
TAG	Triacylglycerol
FA	fatty acid
C16:0	palmitic acid
C18:0	stearic acid
C18:1	oleic acid
C18:2	linoleic acid
C18:3	linolenic acid
C20:0	arachidic acid
C22:0	behenic acid
CLA	conjugated linoleic acid
UFAs	unsaturated fatty acid
SFAs	Saturated fatty acid
*WRI1*	*WRINKLED1*
*FUS3*	*FUSCA3*
*ABI3*	*ABSCISIC ACID INSENSITIVE* 3
*LEC1*	*LEAFY COTYLEDON1*
