# Supplementary figures and images for: Transcriptomic Analysis Reveals Key Genes Involved in Oil and Linoleic Acid Biosynthesis during Artemisia sphaerocephala Seed Development
Source: Int J Mol Sci. 2021 Aug 4;22(16):8369. doi: 10.3390/ijms22168369 (PMC8395072; doi:10.3390/ijms22168369)

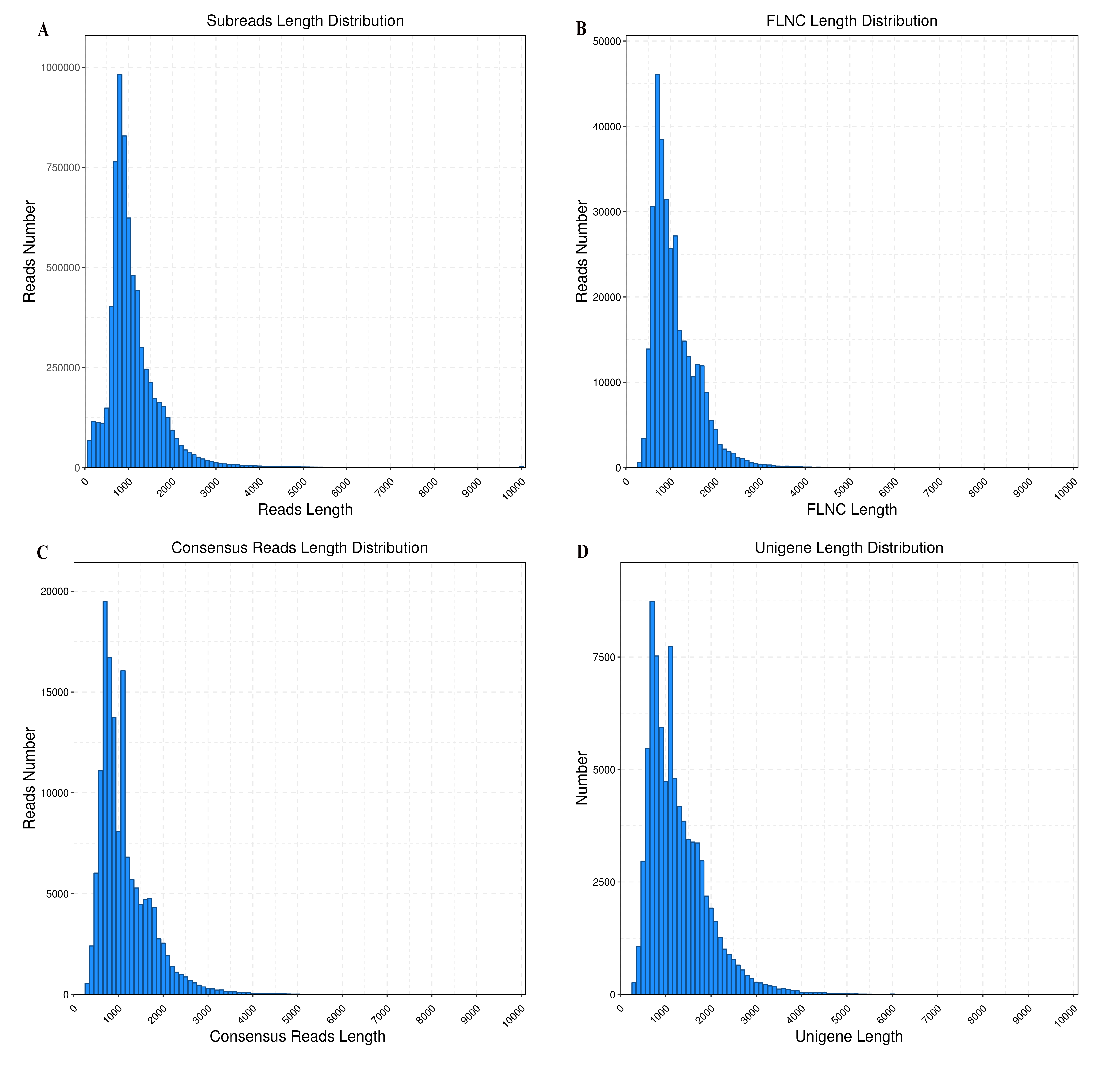

Supplement: Supplementary file 1 [file ijms-22-08369-s001.zip › Figure S1.tif]

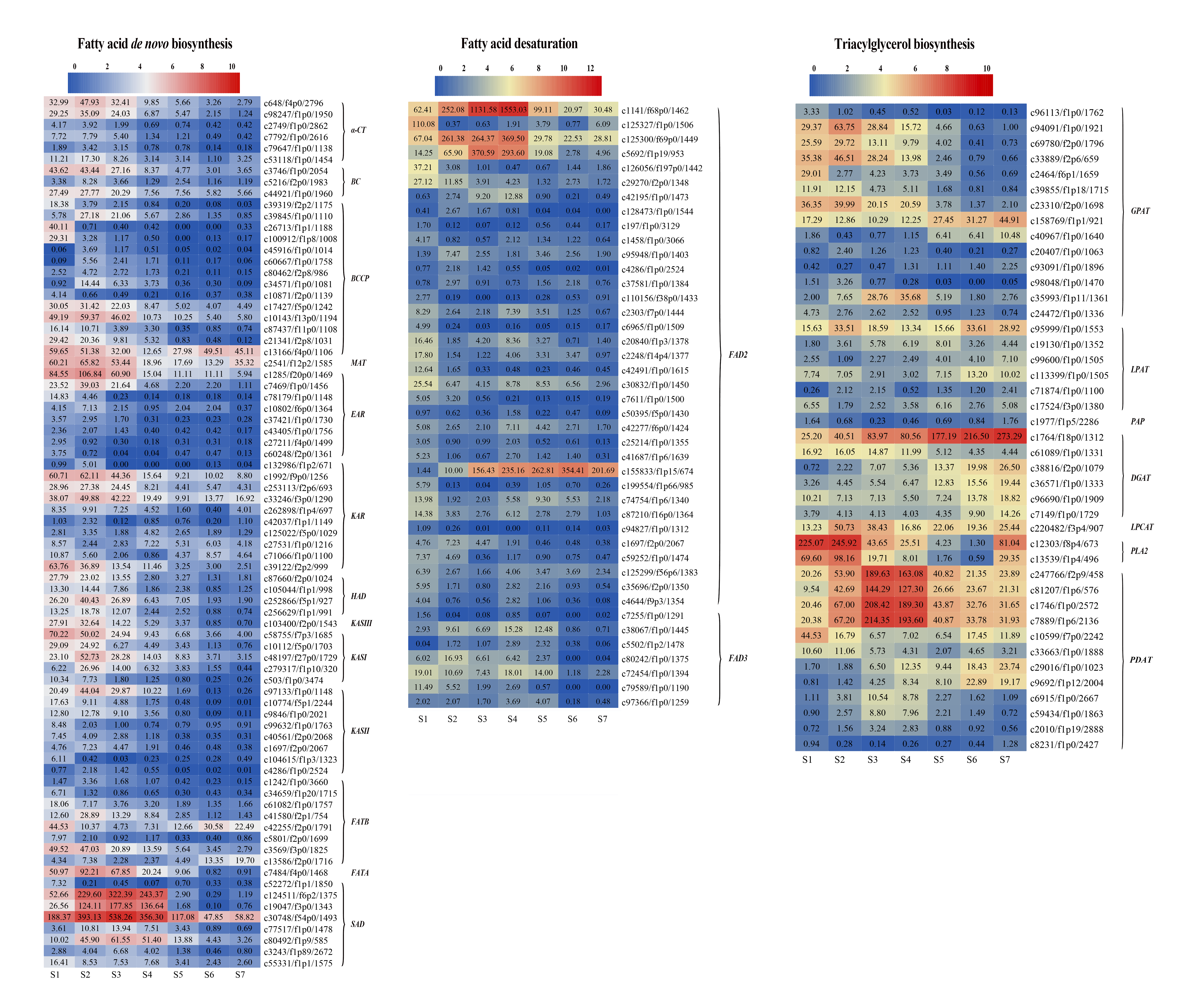

Supplement: Supplementary file 1 [file ijms-22-08369-s001.zip › Figure S2.tif]

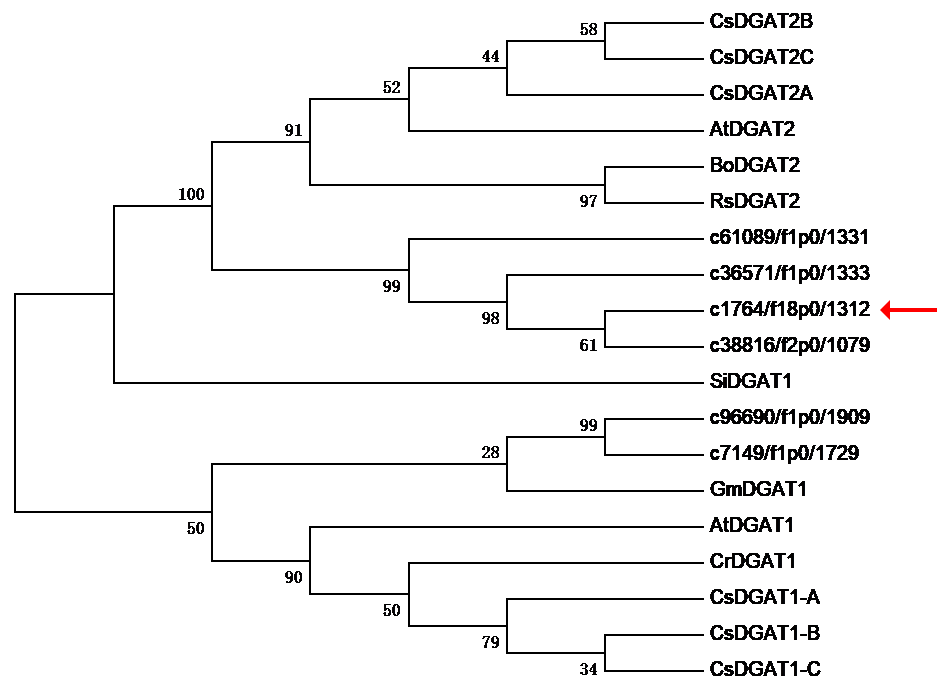

Supplement: Supplementary file 1 [file ijms-22-08369-s001.zip › Figure S3.tif]

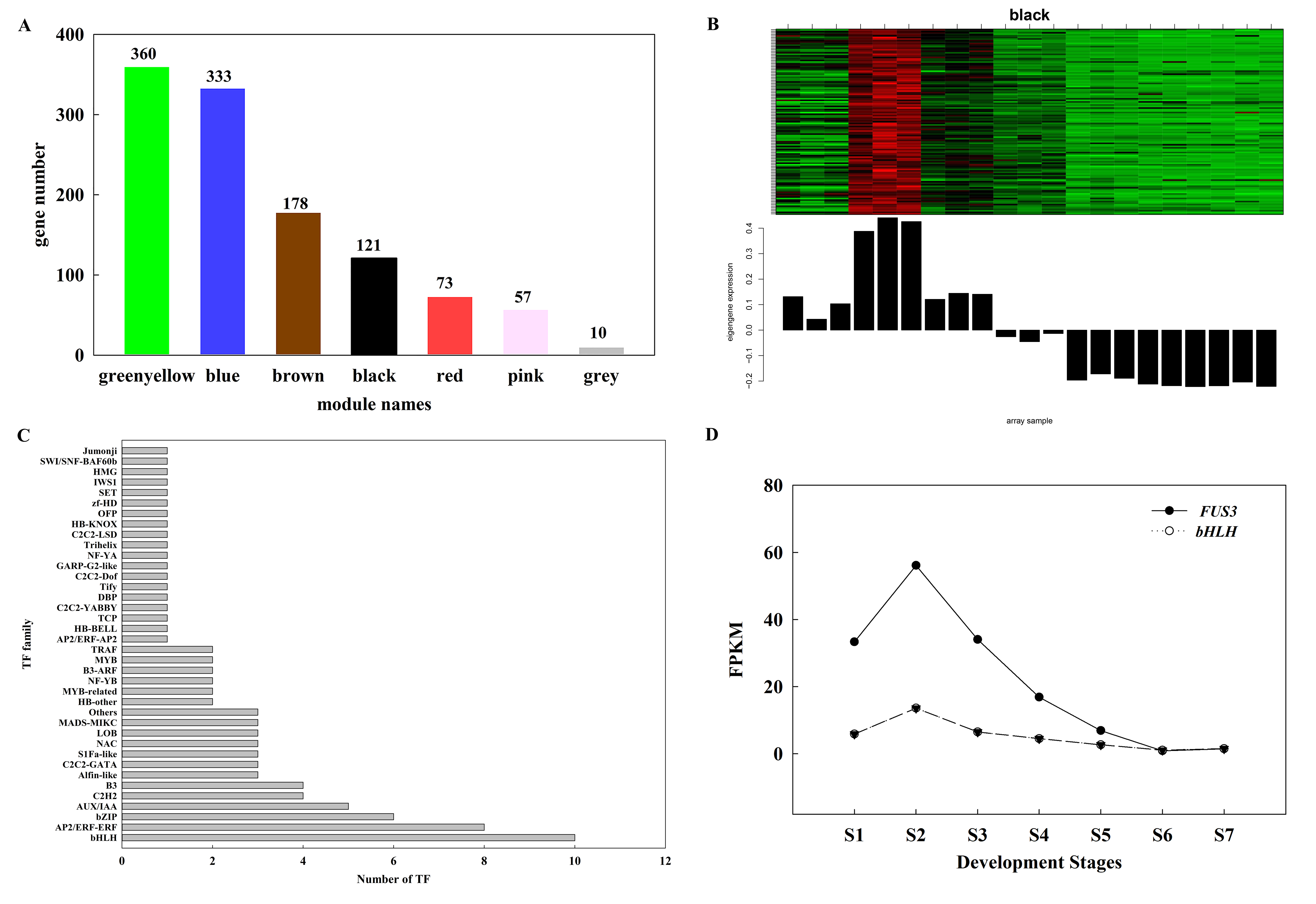

Supplement: Supplementary file 1 [file ijms-22-08369-s001.zip › Figure S4.tif]
